# Impacts of Chemokine (C-X-C Motif) Receptor 2 C1208T Polymorphism on Cancer Susceptibility

**DOI:** 10.1155/2021/8727924

**Published:** 2021-10-14

**Authors:** Jing Zhou, Hao Wu, Quan-Xin Su, Xiao-Kai Shi, Bo-Wen Tang, Cui-Ping Zhao, Hai Wang, Xiao-Ping Chen

**Affiliations:** ^1^Department of Oncology, Affiliated Hospital of Jiangnan University, Hefeng Road 1000, Wuxi 214000, China; ^2^Department of Urology, Changzhou No.2 People's Hospital, 29 Xinglong Road, Changzhou 213003, China; ^3^Department of Geriatrics, Changzhou No.2 People's Hospital, 213000 Changzhou, China; ^4^Department of Oncology, Jintan People's Hospital, Jiangsu University, Changzhou 213002, China

## Abstract

**Background:**

The CXC chemokines belong to a unique family of cytokines that participates in the progression and development of many malignant tumors. Evidence for the relationship between chemokine (C-X-C motif) receptor 2 (CXCR2) C1208T polymorphism and susceptibility to cancer remains inconsistent.

**Methods:**

Odds ratios (ORs), 95% confidence intervals (CIs), and combined analysis were used to investigate the effect of CXCR2 variation on cancer risk. Gene Set Enrichment Analysis (GSEA) and enzyme-linked immunosorbent assay (ELISA) were also used to evaluate the expression of CXCR2 in prostate cancer (PCA).

**Results:**

Across 11 case-control studies, 4,909 cases and 5,884 controls were involved in the current analysis. Individuals with a TT genotype were associated with increased risk of digestive cancer, compared to those with a TC+CC genotype (OR = 1.16, 95%CI = 1.02-1.31, *P* = 0.025). Individuals carrying the TT genotype had a 39% higher risk of urinary cancer than those carrying CC genotype (OR = 1.39, 95%CI = 1.04-1.87, *P* = 0.025). Individuals with a TT genotype showed a 56% augmented breast cancer risk, compared to those with a CC genotype (OR = 1.56, 95%CI = 1.03-2.35, *P* = 0.034). It was found that CXCR2 expression was downregulated in PCA. Compared with PCA subjects carrying the CC genotype, the expression of CXCR2 was decreased in patients with the TT genotype.

**Conclusions:**

The CXCR2 C1208T variation was associated with elevated risk of urinary, breast, and digestive cancer. However, the C1208T polymorphism was correlated with attenuated risk of lung cancer.

## 1. Introduction

Cancer continues to be a major public health problem all over the world [1]. The incidence and mortality of cancer are increasing in both developing and developed countries [2]. Previous studies have shown that there were approximately 14.1 million new cancer subjects and 8.2 million deaths worldwide in 2012 [3]. In China, the number of new cancer patients was more than 4.3 million in 2018 [4]. Late diagnosis, metastasis, and drug resistance lead to a poor survival rate for most malignant tumors [5]. Thus far, specific tumor biomarkers have not been developed for many solid tumors. Therefore, it is necessary to investigate universal molecular markers to predict prognosis and provide targets for cancer patient treatment [6].

Chemokines are a large class of structurally related small protein molecules that play an important role in cell recruitment and migration [7, 8]. Previous studies have shown that CXC chemokines and their receptors are involved in leukocyte migration, angiogenesis, embryogenesis, tumor growth, and metastasis [9–11]. CXC chemokine receptor-2 (CXCR2) is one of the main receptors in the CXC superfamily [12]. It is a seven-transmembrane G protein-coupled receptor (GPCR) often expressed on the cell membrane of endothelial cells, leukocytes, and tumor cells. Previous research indicates that binding of interleukin-8 (IL-8) to CXCR1 or CXCR2 on the cell surface mediates the biological effect of IL-8 [13]. Subsequent studies confirmed that CXCR2, rather than CXCR1, is the main functional chemokine receptor mediating chemokine-induced angiogenesis and endothelial cell chemotaxis [14–16]. The binding of CXCR2 and chemokine induces proliferation, angiogenesis, and invasion of tumor cells [17, 18]. CXCR2 has a high affinity for chemokines and is thought to be associated with the prognosis of several cancers, including colon cancer, hepatocellular carcinoma, and pancreatic cancer [19–23].

Previous studies have shown that genetic variants of CXCR2 may affect the development of cancer by regulating tumor angiogenesis and the antitumor immune response pathway [24, 25]. An et al. revealed that CXCR2 was associated with poor prognosis in patients with nonmetastatic renal clear cell carcinoma and that CXCR2 can be used as a new prognostic factor [26]. However, several other studies demonstrated no positive correlation of CXCR2 with the outcomes of pancreatic carcinoma or esophageal cancer [27, 28]. The CXCR2 C1208T polymorphism has been investigated in several cancers, including those of the prostate, bladder, breast, colon, and lung. However, the relationship between this variant and susceptibility to cancer remains controversial. The purpose of the present study was to comprehensively investigate the association between CXCR2 C1208T polymorphism and cancer risk combined with all eligible case-control studies [29–36]. Furthermore, we used combined analysis to explore the effect of CXCR2 variation. Additionally, Gene Set Enrichment Analysis (GSEA) and enzyme-linked immunosorbent assay (ELISA) were performed to evaluate the expression of CXCR2 in prostate cancer (PCA).

## 2. Materials and Methods

### 2.1. Search Strategy

We conducted an online database search based on Embase, The National Library of Medicine (NLM), Chinese Wanfang, and Google Scholar. The keywords were (‘+1235C/T' OR ‘C1208T' OR ‘CXCR2' OR ‘Chemokine C-X-C motif receptor 2') AND (‘cancer' OR ‘carcinoma') AND (‘polymorphism' OR ‘variant' OR ‘mutant'). The last search update was on May 31, 2021. To increase the number of selected publications, we also retrieved studies by examining the references in published articles.

### 2.2. Inclusion and Exclusion Criteria

A suitable study was included in our analysis when it met all of the following criteria: (a) case-control studies focused on the relationship between the CXCR2 C1208T polymorphism and susceptibility to cancer, (b) studies containing essential genotype information for calculating ORs, (c) control group must be consistent with Hardy-Weinberg equilibrium (HWE) balance, and (d) manuscripts written in English or Chinese. The exclusion criteria were as follows: (a) data for the control group was not available, (b) not enough data to measure the odds ratios (ORs), and/or (c) no relevance to the CXCR2 C1208T polymorphism and risk of cancer.

### 2.3. Data Extraction

Study characteristics were as follows: name of the author, publication year, origin, cancer type, race of population, control source, genetic distribution of CXCR2 variant, sample size of case and control, HWE, and age range of the case and control groups. In the subgroup analysis, PCA and bladder cancer (BLCA) were classified as the urinary cancer group. The digestive system cancer included gastric, esophageal, colon, and rectal cancers. One study focused on Kaposi's sarcoma, and it was classified as “other cancer.”

### 2.4. Statistical Analyses

We applied ORs and 95% confidence intervals (CIs) to investigate the strength of association between the CXCR2 C1208T variant and susceptibility to cancer. Five genetic models were adopted to evaluate the overall ORs. For the CXCR2 C1208T polymorphism, the five models were allelic contrast (T allele vs. C allele), heterozygous comparison (TC vs. CC), homozygous model (TT vs. CC), dominant (TT+TC vs. CC), and recessive (TT vs. TC+CC) model [37, 38]. A *Q* statistic test was used to calculate the heterogeneity among the included studies. A *P* value of heterogeneity (*P*_heterogeneity_) of less than 0.05 indicated heterogeneity among studies. In this case, the random effects method was selected (DerSimonian and Laird). If *P*_heterogeneity_ > 0.05, the fixed effects method was chosen (Mantel–Haenszel) [39, 40]. We performed Fisher's exact test to measure the *P* value of HWE (*P*_HWE_). Studies that were not in accordance with the HWE balance were removed. Studies with a sample size greater than 1000 were classified as large-sample groups. Stratification analysis included cancer type, control source, race, sample size, and quality of studies. Sensitivity analysis for the CXCR2 C1208T mutation was carried out by removing every single study in turn. Publication bias was evaluated by Begg's funnel plot and Egger's test. *P* > 0.05 indicated no evidence of publication bias. All statistical analyses were conducted using STATA software (v11.0, Stata Company, College Station, TX, USA).

### 2.5. Combined Analysis of CXCR2

Minor allele frequencies (MAFs) in global populations were investigated using the HapMap database of the National Center for Biotechnology Information (NCBI) repository (https://www.ncbi.nlm.nih.gov/snp). The gene expression profile of CXCR2 in various cancers was assessed using Gene Expression Profiling Interactive Analysis (GEPIA, http://gepia.cancer-pku.cn/index.html) and The Human Protein Atlas (THPA, https://www.proteinatlas.org/) database. The immune cell infiltration survival curve of PCA, BLCA, and kidney renal clear cell carcinoma (KIRC) patients was evaluated by the Tumor Immune Estimation Resource (TIMER) database (https://cistrome.shinyapps.io/timer). The expression of CXCR2 in PCA was assessed by the Tumor, Normal and Metastatic plot database (https://tnmplot.com/analysis/). The STRING online server was adopted to demonstrate the protein-protein correlation of the CXCR2 protein in *Homo sapiens* (https://string-db.org/cgi/input.pl). GSEA of the transcriptomes in PCA samples was conducted via GSEA software (version 4.1.0, http://software.broadinstitute.org/gsea/index.jsp), a joint project produced by UC San Diego and the Broad Institute. We used GSEA to evaluate the difference of gene expression between the CXCR2 high-expression and low-expression groups in MSigDB library (c2.cp.KEGG.v7.4.symbol.gmt) enrichment. For this analysis, gene set alignment was performed 1000 times. Phenotypic tags were represented by high or low CXCR2 expression values, and all other parameters were default values [41]. A total of 220 PCA patients (confirmed by needle biopsy) were enrolled from Changzhou No.2 People's Hospital and the Affiliated Hospital of Jiangnan University. After signing the written informed consent, each patient donated 2 mL of peripheral blood for the detection of serum CXCR2 by ELISA. The tissue expression of CXCR2 was evaluated by immunohistochemical staining (IHS) in PCA patients from our hospitals. For ELISA analysis, we collected the patient's blood in a standard cube that did not contain anticoagulants. A serum separation tube was used to solidify and centrifuge the sample at 1000 × g (15 min). The serum was immediately taken out for determination and stored evenly or at -80°C. A CUSABIO ELISA kit was used to detect the expression of CXCR2 in the sera of the subjects recruited by our hospitals [42, 43]. For IHS, paraffin sections of PCA samples were embedded in 1% hydrogen peroxide. Then, the slides were washed using a phosphate buffer saline. Goat serum was used to block nonspecific protein interactions. We then incubated the sections with anti-CXCR2 antibody in a concentration of 1 : 200. Diaminobenzidine was used to color the immunoreactive sites brown. The present research was approved by the Ethics Committee of the Affiliated Hospital of Jiangnan University and Ethics Committee of Changzhou No.2 People's Hospital.

## 3. Results

### 3.1. Characteristics of Studies

**Figure 1:**
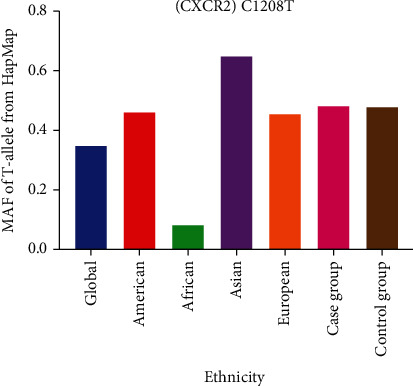
Minor allele frequencies of CXCR2 C1208T polymorphism in reported races and the current analysis.

In total, 11 case-control studies with 4,909 cancer patients and 5,884 control subjects were involved in the current analysis (Table 1). In the subgroup analysis by type of cancer, four studies focused on digestive cancer, three studies on lung cancer, two on breast and urinary system cancer, and one on “other cancer” (Kaposi's sarcoma). In stratified analysis by race, the number of studies on European patients was six, and four studies focused on Asian patients (two on East Asians and two on West Asians). An additional study focused on participants of African descent. Stratification analysis by source of control revealed eight population-based and three hospital-based studies. Stratified analysis by sample size included eight studies with small size and three studies with large size. Moreover, MAFs of the CXCR2 C1208T variation were as follows: Africans, 0.078; Americans, 0.461; Europeans, 0.455; global population, 0.346; and Asians, 0.650. The MAFs in the current study was as follows: case group, 0.484 and control group, 0.479 (Figure 1).

### 3.2. Main Results

**Figure 2:**
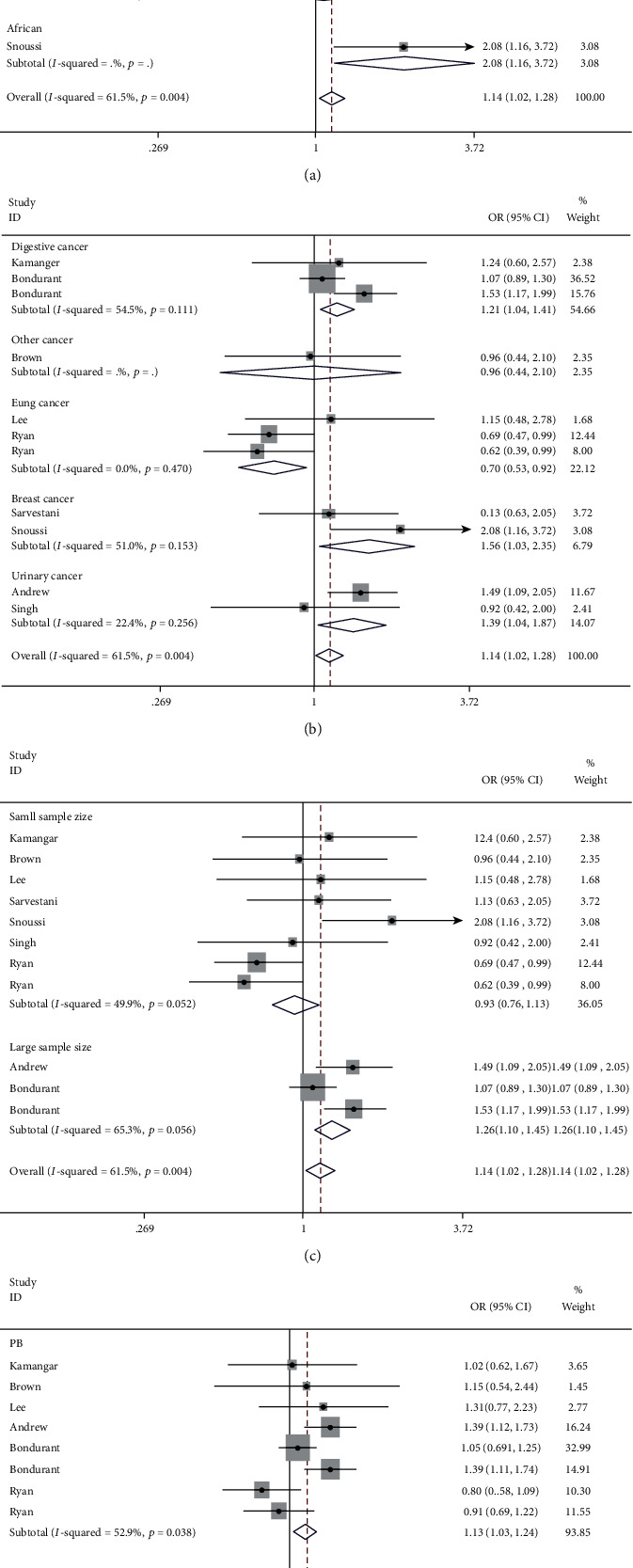
Forest plot of the association between CXCR2 C1208T variant and cancer risk in subgroup analysis by race (a), cancer type (b), sample size (c), and control source (d).

We used ORs and 95% CIs to evaluate the relationship between the CXCR2 C1208T variation and the risk of cancer. Stratified analysis by race did not reveal a significant association of this variant in the European (homozygous comparison, OR = 1.15, 95%CI = 0.89-1.48, *P* = 0.296), East Asian (OR = 0.71, 95%CI = 0.47-1.08, *P* = 0.109), and West Asian groups (OR = 1.05, 95%CI = 0.65-1.68, *P* = 0.845, Figure 2(a)). However, in the subgroup analysis by cancer type, individuals carrying the TT genotype had a 39% higher risk of urinary cancer than those carrying the CC genotype (homozygous comparison, 95%CI = 1.04-1.87, *P* = 0.025, Figure 2(b)). In homozygous comparison, the CXCR2 C1208T variation was also associated with elevated risk of breast (OR = 1.56, 95%CI = 1.03-2.35, *P* = 0.034) and digestive cancer (OR = 1.21, 95%CI = 1.04-1.41, *P* = 0.014). Similar results were evident in a recessive genetic model (breast cancer: OR = 1.60, 95%CI = 1.08-2.38, *P* = 0.020, *I*^2^ = 36.2; digestive cancer: OR = 1.16, 95%CI = 1.02-1.31, *P* = 0.025). For lung cancer, individuals with the TT genotype had a 30% decreased risk compared to those with CC genotype (95%CI = 0.53-0.92, *P* = 0.010). Similar findings were observed in the allelic contrast, heterozygous comparison, and dominant models. In the subgroup analysis by sample size, the results were different between the large- and small-sample studies. In small-sample size studies, individuals with the TT+TC genotype had a 15% decreased risk compared to those with the CC genotype (95%CI = 0.74-0.98, *P* = 0.026). In large-sample size studies, individuals carrying the TC genotype had a 26% increased risk, compared to those with the CC (95%CI = 1.10-1.45, *P* = 0.001, Figure 2(c)). In the subgroup analysis by source of control, the TT genotype was associated with increased risk of cancer in hospital-based studies using the recessive model (OR = 1.47, 95%CI = 1.04-2.09, *P* = 0.030, Figure 2(d)). However, no positive association was revealed in population-based studies (OR = 1.12, 95%CI = 0.96-1.30, *P* = 0.162).

### 3.3. Combined Analysis of CXCR2

**Figure 3:**
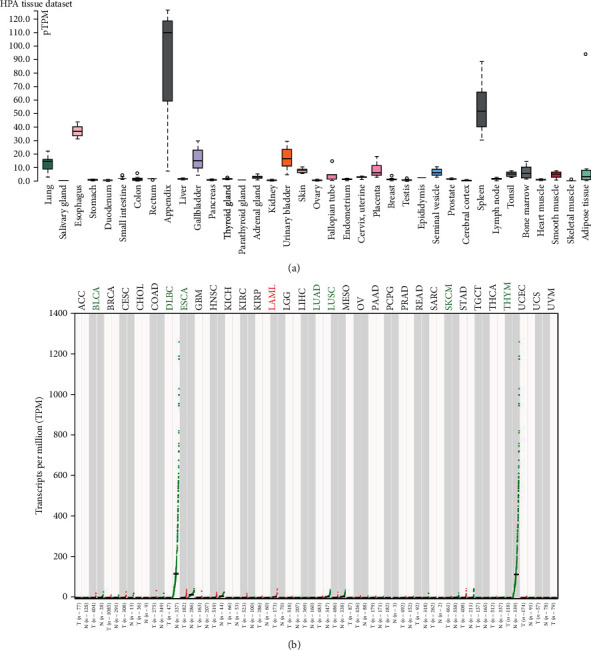
Expression of CXCR2 in human tissues and various cancers. The expression of CXCR2 was downregulated in BLCA, lymphoid neoplasm diffuse large B-cell lymphoma (DLBC), esophageal carcinoma (ESCA), head and neck squamous cell carcinoma (HNSC), lung adenocarcinoma (LUAD), skin cutaneous melanoma (SKCM), and thymoma (Thymoma) (b) subjects. Nevertheless, CXCR2 expression was augmented in patients with acute myeloid leukemia (LAML).

**Figure 4:**
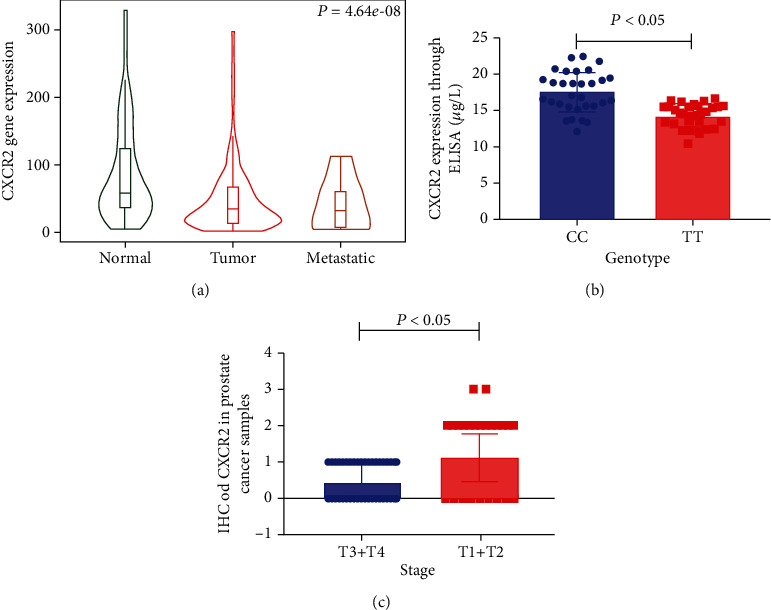
Expression of CXCR2 in PCA. Expression of CXCR2 was downregulated in PCA (a). Compared with PCA subjects carrying the CC genotype, the expression of CXCR2 was decreased in patients with the TT genotype ((b), *P* < 0.05). Expression of CXCR2 was attenuated in more advanced PCA, compared with less advanced patients (T3+T4 versus T1+T2, *P* < 0.05, (c)).

We used the THPA database to evaluate the expression of CXCR2 in human tissues. CXCR2 was mainly expressed in the human appendix, spleen, bladder, and esophagus (Figure 3(a)). We further used GEPIA database to explore the CXCR2 expression in various cancers. CXCR2 expression was downregulated in BLCA, lymphoid neoplasm diffuse large B-cell lymphoma (DLBC), esophageal carcinoma (ESCA), head and neck squamous cell carcinoma (HNSC), lung adenocarcinoma (LUAD), skin cutaneous melanoma (SKCM), and thymoma (Thymoma) (Figure 3(b)) subjects. Nevertheless, CXCR2 expression was augmented in patients with acute myeloid leukemia (LAML). Moreover, ELISA was employed to assess the serum expression of CXCR2 in PCA patients recruited from our hospitals. Compared with PCA subjects carrying the CC genotype, the expression of CXCR2 was decreased in patients with the TT genotype (Figure 4(b), *P* < 0.05). Furthermore, we used IHS analysis to verify the results of bioinformatical findings. As shown in Figure 4(c), the expression of CXCR2 was attenuated in more advanced PCA, compared with less advanced patients (T3+T4 versus T1+T2, *P* < 0.05). The expression of CXCR2 was decreased in PCA participants, relative to that in normal counterparts (*P* < 0.05, Figure 4(a)).

**Figure 5:**
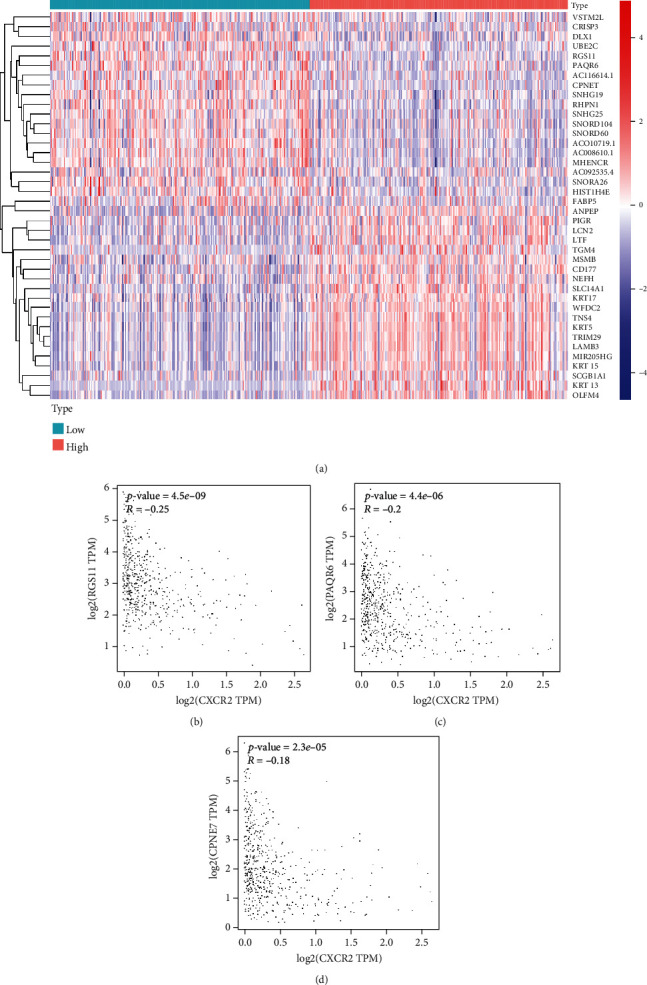
Gene-gene correlation of CXCR2 in PCA patients. (a) Differential expressed genes between the high and low expressions of CXCR2. The most correlated genes were RGS11 (regulator of G protein signaling 11, (b)), PAQR6 (progestin and adipoQ receptor family member 6, (c)), and CPNE7 (copine 7, (d)).

**Figure 6:**
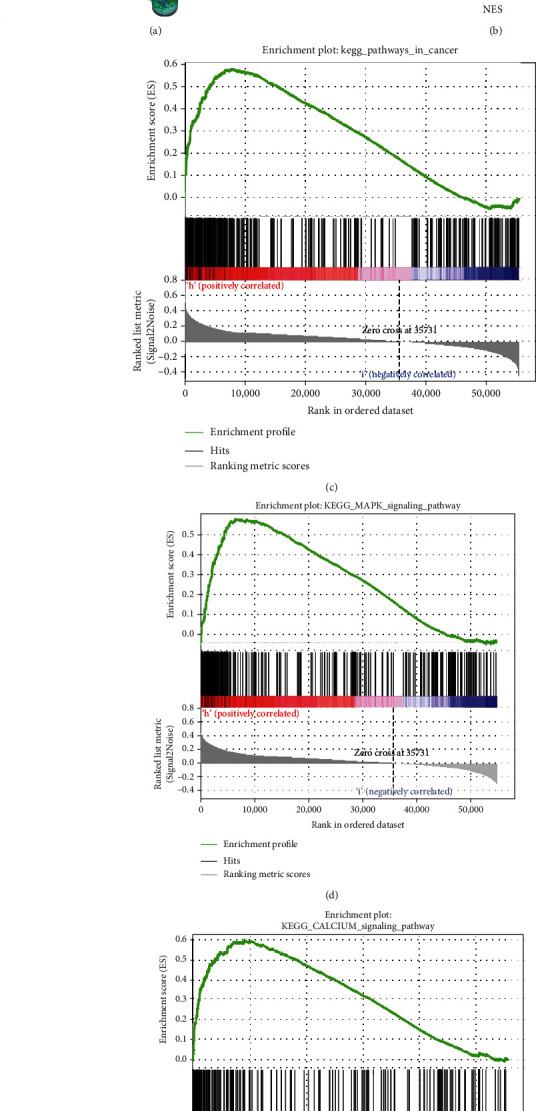
The expression and correlation of CXCR2 evaluated by the Gene Set Enrichment Analysis (GSEA) and STRING tools. More than 10 proteins can be involved in the interaction with CXCR2 protein. They were interleukin-8 (CXCL8), C-X-C motif chemokine 5 (CXCL5), growth-regulated alpha protein (CXCL1), C-X-C motif chemokine 2 (CXCL2), platelet basic protein (PPBP), C-X-C motif chemokine 6 (CXCL6), C-X-C motif chemokine 3 (CXCL3), stromal cell-derived factor 1 (CXCL12), C-X-C chemokine receptor type 1 (CXCR1), and C-C motif chemokine 5 (CCL5) (a). Plot of normalized enrichment score (NES) versus false positive rate (FDR) (b). GSEA showed evidence that the expression of CXCR2 is correlated with pathways in cancer ((c), *P* < 0.05). Signaling pathways, including mitogen-activated protein kinase (MAPK) signaling (d) and calcium signaling system (e), were associated with the high expression of CXCR2.

**Figure 7:**
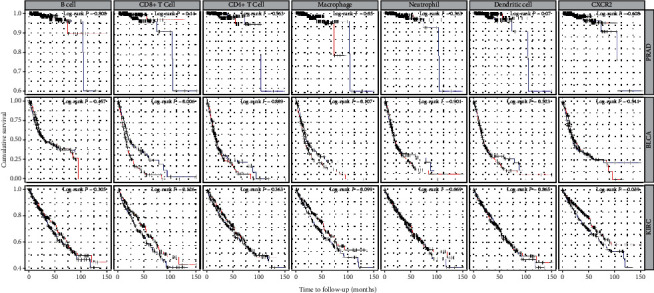
Immune cell infiltration survival curve of PCA, BLCA, and KIRC patients evaluated by TIMER database. High degree of infiltration was marked with a red line. Low degree of infiltration was marked with a blue line.

We used R language to investigate the gene-gene correlation of CXCR2 in PCA. The heatmap plot was shown in Figure 5(a). The most correlated genes include regulator of G protein signaling 11 (*RGS11*, Figure 5(b)), progestin and adipoQ receptor family member 6 (*PAQR6*, Figure 5(c)), and copine 7 (*CPNE7*, Figure 5(d)). We further employed the STRING database to investigate the protein-protein correlations of CXCR2. More than 10 proteins were involved in interacting with the CXCR2 protein (Figure 6(a)). They were interleukin-8 (CXCL8), C-X-C motif chemokine 5 (CXCL5), growth-regulated alpha protein (CXCL1), C-X-C motif chemokine 2 (CXCL2), platelet basic protein (PPBP), C-X-C motif chemokine 6 (CXCL6), C-X-C motif chemokine 3 (CXCL3), stromal cell-derived factor 1 (CXCL12), C-X-C chemokine receptor type 1 (CXCR1), and C-C motif chemokine 5 (CCL5). We further performed GSEA to investigate potential associated signaling pathways correlated with CXCR2 expression. Plot of normalized enrichment score (NES) versus false positive rate (FDR) was indicated in Figure 6(b). The GSEA showed evidence that the expression of CXCR2 is correlated with pathways in cancer (Figure 6(c), *P* < 0.05). Signaling pathways, including mitogen-activated protein kinase (MAPK) signaling (Figure 6(d)) and calcium signaling system (Figure 6(e)), were associated with a high expression of CXCR2. Furthermore, we used the TIMER database to evaluate the prognostic relevance of immune cell infiltration in PCA, BLCA, and KIRC samples. The survival time of BLCA patients with high CD8+ T cells was significantly shorter than that of BLCA patients with low infiltration (*P* = 0.006). The survival time of KIRC patients with a high expression of CXCR2 was significantly longer than that in the low CXCR2 group (*P* = 0.039, Figure 7).

### 3.4. Sensitivity Analysis and Publication Bias

We performed a sensitivity analysis to evaluate the effect of each single study on overall ORs. Publication bias was investigated using Begg's funnel plots and Egger's tests. As shown in Supplemental Figure 1A, no single study was found to have a significant impact on the ORs when assessing the CXCR2 C1208T polymorphism. Begg's funnel plots (Supplemental Figure 1C, *P* > 0.05) and Egger's tests (Supplemental Figure 1B, *P* > 0.05) also revealed no evidence of publication bias among the studies on the CXCR2 variants.

## 4. Discussion

Carcinoma remains a significant health issue worldwide, even though a large number of previous studies have explored the susceptibility and high-risk factors of malignant tumors. However, specific markers for several cancers have not been identified up to now [44, 45]. It is necessary to investigate universal molecular markers to form a prognosis for the treatments of cancer patients. Previous studies showed that the expression of CXC chemokine receptors, including CXCR2, is associated with necrosis and development of several cancers [23, 46, 47]. Moreover, CXCR2 acts as an autocrine or paracrine growth factor to induce tumor invasion and migration [48]. Genetic polymorphisms of CXCR2 may influence the function of the protein by regulating gene expression. Several previous studies have investigated the correlation of CXCR2 mutations with risk of cancer [32–38]. However, the conclusions of these studies were not consistent. Ryan et al. [36] evaluated the CXCR2 polymorphism in Indians and revealed that C1208T was associated with an increased risk of bladder cancer (*P* = 0.003, OR = 1.29). Peng et al. [37] assessed the CXCR2 variant in two independent populations (European and Asian) and found that C1208T was associated with a reduced risk of lung cancer. In 2017, Yang et al. [48] performed a meta-analysis and indicated that CXCR2 expression in tumor tissues was correlated with poor prognosis in solid tumor patients. In 2018, Qiao et al. [49] conducted another meta-analysis and revealed that CXCR2 was an unfavorable predictor of overall survival and recurrence-free survival in patients with cancers, except for digestive cancer. However, these researchers did not evaluate the association between the CXCR2 C1208T polymorphism and susceptibility to cancer, which remains controversial. Recently, Zhu et al. conducted a pooled analysis and found that interleukin-8 receptor B rs1126579 C>T variation may be correlated with susceptibility to cancer [50]. However, they did not include all the available data on this gene polymorphism. In the current study, we pooled 14 eligible case-control studies on the CXCR2 C1208T variant, comprising 4,909 cancer cases and 5,884 controls. Our study revealed a significant association between the CXCR2 C1208T polymorphism and risk of cancer. The CXCR2 gene variation played a different role in various tumors.

In the stratified analysis by cancer type, we observed that the C1208T variant was correlated with increased risk of urinary, breast, and digestive cancers. For lung cancer, individuals with the TT genotype had a 30% decreased risk compared to those with the CC genotype, consistent with the reports in previous studies by Peng et al. [37]. In the subgroup analysis by race, no positive results were demonstrated in participants of European or African descent. One possible reason may be that the number of studies on Africans included in our analysis was relatively small. However, results from Bondurant et al. [34] indicate that CXCR2 C1208T polymorphism is associated with increased breast cancer risk in African patients. As described in the subgroup analysis by sample size, the results derived from the large- and small-sample studies may be different. Further research with large-sample sizes on the CXCR2 C1208T variation in African population is warranted in the future. In addition, we used combined analysis to explore the expression of CXCR2 in urinary cancer according to the race of participants. CXCR2 expression was downregulated in BLCA patients of European and African American descent. The expression of CXCR2 was also downregulated in PCA patients of European descent. Expression profiles of CXCR2 in PCA patients of Asian descent were not available from the online database. We used ELISA to investigate the serum expression of CXCR2 in needle biopsy confirmed PCA patients enrolled in our centers. It was observed that the expression of CXCR2 was decreased in PCA participants with the TT genotype, consistent with results derived from the pooled analysis. In addition, CXCR2 expression was decreased in PCA participants, compared with controls. Our results show that patients with advanced PCA had lower levels of CXCR2, compared with early-stage patients. It is suggested that the detection of CXCR2 may provide guidance for the prognosis of patients with PCA.

We further used GSEA to explore the signaling pathways potentially correlated with CXCR2 expression. Signaling pathways, such as MAPK signaling and calcium signaling system, were associated with high CXCR2 expression. Moreover, we used the TIMER database to evaluate the prognostic relevance of immune cell infiltration in PCA, BLCA, and KIRC samples. The survival time of BLCA patients with high CD8+ T cells was significantly shorter than that of BLCA patients with low infiltration. The survival time of KIRC patients with high expression of CXCR2 was significantly longer than that of the low CXCR2 group. There are, however, several limitations to the current analysis. First, there is only one case-control study on participants of African descent based on the inclusion criteria. Future studies with large-sample sizes focused on African populations are warranted. Second, the sample size of the included studies on the CXCR2 C1208T polymorphism remains insufficient. The number of studies on tumors including KIRC, PCA, and BLCA is quite limited. Third, we revealed that the CXCR2 C1208T polymorphism may be associated with increased risk of PCA. Further research is warranted to confirm whether the CXCR2 C1208T mutation has an impact on CXCR2 expression in PCA. As a single variation cannot have a huge impact on the development of cancer, it is necessary to investigate the gene-gene or gene-environment interactions in future research.

In conclusion, the present study summarizes all eligible genetic data for correlation between the CXCR2 C1208T variant and risk of cancer. Our study revealed that the CXCR2 C1208T polymorphism is associated with increased risk of urinary, breast, and digestive cancers. CXCR2 C1208T may also be associated with risk of PCA. Finally, CXCR2 expression was negatively correlated with the degree of PCA malignancy.

## Figures and Tables

**Table 1:** Study characteristics of CXCR2 C1208T polymorphism in the present analysis.

Author C1208T	Year	Origin	Type	Race	Source	Genotype	Case						Control					
							Age (y)	TT	TC	CC	Total	HWE	Age (y)	TT	TC	CC	Total	HWE
Kamangar	2006	Finland	GC	European	PB	TaqMan	Mean 58.5	36	61	15	112	0.172	59 (50-69)	66	108	34	208	0.359
Brown	2006	Italy	KS	European	PB	TaqMan	72 (29-91)	14	40	79	133	0.016	75 (37-92)	16	69	87	172	0.666
Lee	2007	China	LC	East Asian	PB	PCR	NA	55	47	13	115	0.542	NA	44	51	12	107	0.628
Sarvestani	2007	Iran	BC	West Asian	HB	PCR	49.4 (28-85)	27	85	106	218	0.131	NA	27	114	120	261	0.992
Andrew	2009	USA	BLCA	European	PB	GoldenGate	64 (25-74)	250	255	84	589	0.149	NA	299	414	150	863	0.745
Snoussi	2010	Tunisia	BC	African	HB	PCR	48.0 ± 24.0	47	167	195	409	0.222	48.0 ± 14.9	18	128	155	301	0.207
Bondurant	2013	USA	CC	European	PB	GoldenGate	30-79	343	794	417	1554	0.340	NA	411	1009	536	1956	0.112
Bondurant	2013	USA	RC	European	PB	GoldenGate	30-79	201	359	192	752	0.216	NA	199	470	290	959	0.736
Singh	2014	India	BLCA	West Asian	HB	PCR	58.5 ± 12.4	15	73	112	200	0.520	56.8 ± 10.8	14	90	96	200	0.247
Ryan	2015	USA	LC	European	PB	iPlexGold	66.6 ± 10.0	90	215	138	443	0.708	67.4 ± 8.5	115	238	121	474	0.924
Ryan	2015	Japan	LC	East Asian	PB	TaqMan	59.0 ± 8.0	170	160	54	384	0.104	59.0 ± 8.0	178	170	35	383	0.537

BC: breast cancer; BLCA: bladder cancer; BT: biliary tract; CC: colon cancer; GC: gastric cancer; EC: esophageal cancer; KS: Kaposi sarcoma; LC: lung cancer; NA: not available; RC: rectal cancer.

## Data Availability

All the data in this study can be provided by corresponding authors if the request is reasonable.

## Supplementary Materials

Supplementary Materials Supplemental Figure 1: publication bias of the current study assessed by sensitivity analysis, Begg's funnel plot, and Egger's test. Sensitivity analysis of CXCR2 C1208T showed that a single study would not have an impact on the significance of ORs (A). Begg's funnel (C) and Egger's plot (B) analyses also indicated no evidence of publication bias. Supplemental Table 1: stratified analysis of CXCR2 C1208T variation on the likelihood of cancer.
