# Supplementary material for: Impacts of Chemokine (C-X-C Motif) Receptor 2 C1208T Polymorphism on Cancer Susceptibility
Source: J Immunol Res. 2021 Oct 14;2021:8727924. doi: 10.1155/2021/8727924 (PMC8531794; doi:10.1155/2021/8727924)
Supplement: Supplementary Materials — Supplemental Figure 1: publication bias of the current study assessed by sensitivity analysis, Begg's funnel plot, and Egger's test. Sensitivity analysis of CXCR2 C1208T showed that a single study would not have an impact on the significance of ORs (A). Begg's funnel (C) and Egger's plot (B) analyses also indicated no evidence of publication bias. Supplemental Table 1: stratified analysis of CXCR2 C1208T variation on the likelihood of cancer. [file 8727924.f1.doc]

**Impacts of chemokine (C-X-C motif) receptor 2 C1208T polymorphism on cancer susceptibility**

Jing Zhou^1*^, Hao Wu^2*^, Quan-Xin Su ^2*^, Xiao-Kai Shi ^2*^, Bo-Wen Tang ^2*^, Cui-Ping Zhao ^3^, Hai Wang ^4^, Xiao-Ping Chen ^1^

*^1^ Department of Oncology, Affiliated Hospital of Jiangnan University, Hefeng Road 1000, Wuxi 214000, China.*

*^2^ Department of Urology, Changzhou No.2 People's Hospital, 29 Xinglong Road, Changzhou 213003, China.*

^3^ *Department of Geriatrics, Changzhou No.2 People's Hospital, 213000 Changzhou, China.*

^4^ *Department of Oncology, Jintan People's Hospital, Jiangsu University, Changzhou 213002, China.*

*^*^Equal contributors.*

**Address correspondence to:** Cui-Ping Zhao, Department of Geriatrics, Changzhou No.2 People's Hospital, Changzhou 213003, Jiangsu Province, China. E-mail: lnyxk2021@163.com. Hai Wang, Department of Oncology, Jintan People's Hospital, Jiangsu University, Changzhou 213002, China. E-mail: wh81320@163.com; Xiao-Ping Chen, Department of Oncology, Affiliated Hospital of Jiangnan University, Hefeng Road 1000, Wuxi 214000, China. E-mail: 1165079792@qq.com.

**Supplemental Fig. 1 Publication bias of the current study assessed by sensitivity analysis, Begg's funnel plot, and Egger’s test.** Sensitivity analysis of CXCR2 C1208T showed that a single study would not have an impact on the significance of ORs (Figure A). Begg's funnel (Figure C) and Egger’s plot (Figure B) analysis also indicated no evidence of publication bias.

**
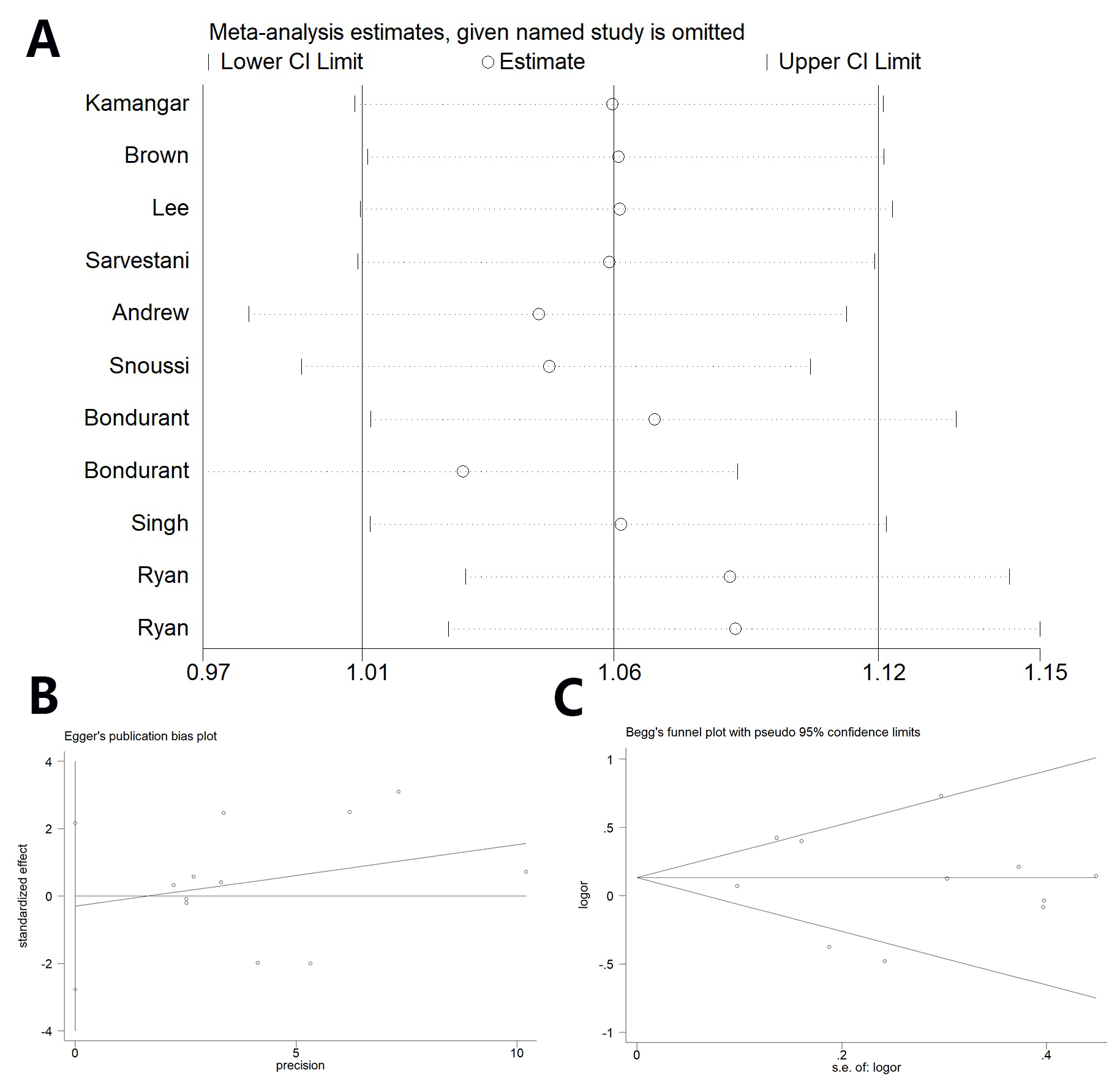
**

**Supplemental Table 1 Stratified analysis of CXCR2 C1208T variation on the likelihood of cancer.**

| Variables | N | Case/ | OR(95%CI) *P*_heter_  *P* | OR(95%CI) *P*_heter_  *P* | OR(95%CI) *P*_heter_  *P* | OR(95%CI) *P*_heter_  *P* | OR(95%CI) *P*_heter_ *P* |
| --- | --- | --- | --- | --- | --- | --- | --- |
| **C1208T** |  | Control | Allelic | Heterozygous | Homozygous | Dominant | Recessive |
| **Total** | 11 | 4909/5884 | 1.03(0.93-1.15) 0.002 0.562 | 0.96(0.87-1.05) 0.131 0.365 | 1.12(0.90-1.39) 0.004 0.322 | 0.96(0.83-1.12) 0.015 0.634 | 1.16(1.00-1.34) 0.040 0.051 |
| **Race** |  |  |  |  |  |  |  |
| EA | 2 | 499/490 | 0.91(0.76-1.10) 0.176 0.340 | 0.66(0.43-1.00) 0.515 0.050 | 0.71(0.47-1.08) 0.222 0.109 | 0.68(0.46-1.02) 0.323 0.060 | 0.99(0.77-1.27) 0.240 0.948 |
| European | 6 | 3583/4632 | 1.05(0.92-1.21) 0.002 0.467 | 1.00(0.90-1.12) 0.169 0.934 | 1.15(0.89-1.48) 0.010 0.296 | 1.03(0.85-1.24) 0.025 0.775 | 1.14(0.95-1.37) 0.032 0.156 |
| WA | 2 | 418/461 | 0.91(0.74-1.12) 0.410 0.391 | 0.77(0.58-1.02) 0.499 0.070 | 1.05(0.65-1.68) 0.675 0.845 | 0.82(0.63-1.06) 0.430 0.132 | 1.17(0.74-1.84) 0.790 0.498 |
| African | 1 | 409/301 | 1.25(0.99-1.58) - 0.058 | 1.04(0.76-1.42) - 0.819 | 2.08(1.16-3.72) - 0.014 | 1.17(0.86-1.57) - 0.315 | 2.04(1.16-3.59) - 0.013 |
| **Type** | |  |  |  |  |  |  |
| DC | 3 | 2418/3123 | 1.10(1.02-1.18) 0.097 0.018 | 1.06(0.94-1.21) 0.561 0.349 | 1.21(1.04-1.41) 0.111 0.014 | 1.11(0.98-1.25) 0.280 0.099 | 1.16(1.02-1.31) 0.143 0.025 |
| LC | 3 | 942/964 | 0.87(0.76-0.99) 0.301 0.033 | 0.74(0.58-0.95) 0.633 0.018 | 0.70(0.53-0.92) 0.470 0.010 | 0.73(0.58-0.92) 0.566 0.008 | 0.91(0.75-1.11) 0.282 0.342 |
| BC | 2 | 627/562 | 1.13(0.95-1.35) 0.192 0.166 | 0.96(0.75-1.22) 0.415 0.711 | 1.56(1.03-2.35) 0.153 0.034 | 1.05(0.83-1.32) 0.278 0.684 | 1.60(1.08-2.38) 0.211 0.020 |
| UC | 2 | 789/1063 | 1.05(0.70-1.57) 0.018 0.832 | 0.93(0.73-1.19) 0.081 0.577 | 1.39(1.04-1.87) 0.256 0.025 | 0.97(0.57-1.68) 0.026 0.924 | 1.36(1.11-1.68) 0.524 0.003 |
| Other | 1 | 133/172 | 0.83(0.58-1.18) - 0.299 | 0.64(0.39-1.05) - 0.075 | 0.96(0.44-2.10) - 0.926 - | 0.70(0.44-1.11) - 0.126 | 1.15(0.54-2.44) - 0.722 |
| **Source of control** | | | |  |  |  |  |
| PB | 8 | 4082/5122 | 1.03(0.91-1.17) 0.001 0.627 | 0.98(0.88-1.09) 0.105 0.678 | 1.06(0.83-1.36) 0.003 0.637 | 0.97(0.81-1.17) 0.012 0.770 | 1.12(0.96-1.30) 0.038 0.162 |
| HB | 3 | 827/762 | 1.05(0.90-1.22) 0.099 0.535 | 0.88(0.71-1.08) 0.306 0.231 | 1.39(0.97-2.00) 0.186 0.073 | 0.95(0.78-1.16) 0.158 0.648 | 1.47(1.04-2.09) 0.310 0.030 |
| **Size** | | | |  |  |  |  |
| Small | 8 | 2014/2106 | 0.94(0.86-1.03) 0.116 0.201 | 0.82(0.71-0.95) 0.433 0.008 | 0.93(0.76-1.13) 0.052 0.462 | 0.85(0.74-0.98) 0.198 0.026 | 1.03(0.88-1.20) 0.180 0.740 |
| Large | 3 | 2895/3778 | 1.16(1.01-1.33) 0.028 0.033 | 1.06(0.94-1.20) 0.631 0.323 | 1.26(1.10-1.45) 0.056 0.001 | 1.13(1.01-1.26) 0.210 0.041 | 1.22(1.09-1.37) 0.065 <0.001 |

BC: Breast cancer; DC: Digestive cancer; EA: East Asian; HB: Hospital based; WA: West Asian; N: Number of included studies; LC: Lung cancer; PCR: polymerase chain reaction; UC: Urinary cancer; PB: Population based. *P*_heter_: *P* value of heterogeneity test.
